# Phenotypic Variation Patterns in *Oecomys catherinae* (Rodentia: Sigmodontinae): Craniodental Morphometric Analysis and Its Relationship with Latitudinal Variation in the Atlantic Forest and Cerrado Biomes

**DOI:** 10.3390/ani15152200

**Published:** 2025-07-26

**Authors:** Paola Santos da Mata, Thiago dos Santos Cardoso, Cibele Rodrigues Bonvicino, Roberto do Val Vilela

**Affiliations:** 1 Departamento de Genética, Instituto de Biologia, Centro de Ciências da Saúde, Universidade Federal do Rio de Janeiro, Ilha do Fundão—Cidade Universitária, Rio de Janeiro 21941-617, RJ, Brazil; 2 Laboratório de Biologia e Parasitologia de Mamíferos Silvestres Reservatórios, Instituto Oswaldo Cruz (IOC/Fiocruz), Avenida Brasil 4365, Manguinhos, Rio de Janeiro 21040-900, RJ, Brazil; thgo_cardoso@yahoo.com.br (T.d.S.C.); cibelebonvicino@gmail.com (C.R.B.)

**Keywords:** neotropical rodents, morphological divergence, phenotypic plasticity, latitudinal gradients

## Abstract

The arboreal rodent *Oecomys catherinae*, which has a wide geographic distribution across Brazilian biomes, provides a model for investigating environmental influences on morphological variation. We assessed craniodental differences between the Atlantic Forest and Cerrado biomes, as well as along latitudinal gradients, using 45 specimens from 18 localities. Linear morphometric analyses (21 measurements) revealed no significant sexual dimorphism, allowing for pooled analyses. Principal Component Discriminant Analysis (DAPC) confirmed significant morphological divergence between biomes (72% accuracy, *p* < 0.01). We identified bony palate length (BPL) as the most discriminating variable, with higher values in the Atlantic Forest, suggesting a possible dietary adaptation in response to ecological pressures. Latitudinal effects were modest (adjusted R^2^ = 0.05) although significant (F_1,43_ = 3.63; *p* = 0.03), with southern populations exhibiting larger cranial dimensions than northern ones. We conclude that biome type and latitude played important roles in shaping cranial morphology in *O. catherinae* populations.

## Simple Summary

Understanding how animals adapt to natural environments reveals essential physical and behavioral traits for survival. We examined skull shape variation in the arboreal rodent *Oecomys catherinae* across two Brazilian biomes, the Atlantic Forest and Cerrado, and its relationship with latitude. Through cranial and dental measurements of 45 specimens from scientific collections, no significant differences were observed between males and females. However, cranial morphology differed between biomes, likely due to dietary adaptations and ecological pressures. Southern populations exhibited slightly larger cranial dimensions than northern counterparts, consistent with Bergmann’s rule (which posits that larger body sizes occur in colder climates). These results demonstrate how environmental pressures shape physical traits in small mammals. Further research is required to determine whether these differences reflect genetic adaptations or plastic responses (non-heritable changes induced by local environments).

## 1. Introduction

The genus *Oecomys* (Rodentia: Cricetidae) comprises semi-arboreal rodents with morphological and behavioral adaptations for efficiently utilizing arboreal strata in humid tropical forests [1]. These nocturnal species rely on vegetation for locomotion, feeding, and nesting, occupying both arboreal strata and the ground [2]. Morphological traits such as reduced limb proportions reflect this arboreal specialization, making them the most anatomically adapted Oryzomyini to this lifestyle [3].

The taxonomic classification of *Oecomys* is challenging due to high interspecific similarity, requiring integrative analyses of morphological and genetic traits [4,5]. This complexity has left many species unresolved, underscoring the need for further studies [6,7,8]. Additionally, the lack of long-term autecological studies represents a significant gap in understanding their biology and environmental interactions [2]. Among the species of *Oecomys*, widely distributed across South America, *Oecomys catherinae* (Thomas, 1909) [9] stands out due to its broad geographic range in Brazil, occurring along the eastern region from the state of Santa Catarina to Paraíba [2,10,11]. *Oecomys catherinae* occupies multiple biomes, including the Amazon, Atlantic Forest, and Cerrado, being one of the most widely distributed taxa in Brazil [12].

Phenotypic variation in small mammals, such as those of the genus *Oecomys*, can be measured through linear or geometric morphometric analyses of craniodental structures [13,14]. When correlated with environmental gradients (e.g., latitude, habitat heterogeneity), these variations may reflect mechanisms like phenotypic plasticity or local adaptations [15]. Although studies have demonstrated these associations in Amazonian Sigmodontinae rodents [1], the underlying mechanisms (plasticity versus adaptation) remain poorly investigated in widely distributed Oryzomyini such as *O. catherinae*.

The Atlantic Forest and Cerrado, as biodiversity hotspots [16], provide contrasting environmental gradients for *O. catherinae*. The Atlantic Forest exhibits remarkable altitudinal (0–2700 m) and latitudinal (3–30° S) heterogeneity, encompassing humid forests and semi-humid formations across diverse soils [17,18]. In contrast, the Cerrado combines seasonal savannas (cerrado sensu stricto), gallery forests, veredas, and rocky outcrop vegetation (campos rupestres) on acidic, oligotrophic soils under a seasonal tropical climate [19,20].

We hypothesize that craniodental morphology in *O. catherinae* varies significantly along latitudinal variation and between biomes, reflecting both local adaptations and phenotypic plasticity to environmental variation. To test this, we conducted morphometric analyses on specimens from the Atlantic Forest and Cerrado biomes, correlating morphological variation with environmental parameters (latitude and biome type) using multivariate statistical approaches. Understanding these patterns advances knowledge of *O. catherinae* biology and the mechanisms of adaptation and phenotypic plasticity in neotropical small mammals. Furthermore, this study reinforces the importance of integrating morphometric and ecological approaches to elucidate how environmental factors may shape phenotypic diversity across geographic scales and ecological contexts.

## 2. Materials and Methods

### 2.1. Sampling and Specimens Localities

Morphometric studies were conducted using small rodent specimens from the scientific collections of the Museu Nacional, Universidade Federal do Rio de Janeiro (MN/UFRJ), and the Coleção Integrada de Mamíferos Reservatórios Silvestres, Instituto Oswaldo Cruz, Fundação Oswaldo Cruz (COLMASTO/IOC/FIOCRUZ). Forty-five specimens of *O. catherinae* were selected based on their documented geographic origin, availability of complete biometric data, and proper craniodental preservation (Table S1: Geographic location, sex, and age classification of *O. catherinae* specimens).

**Figure 1:**
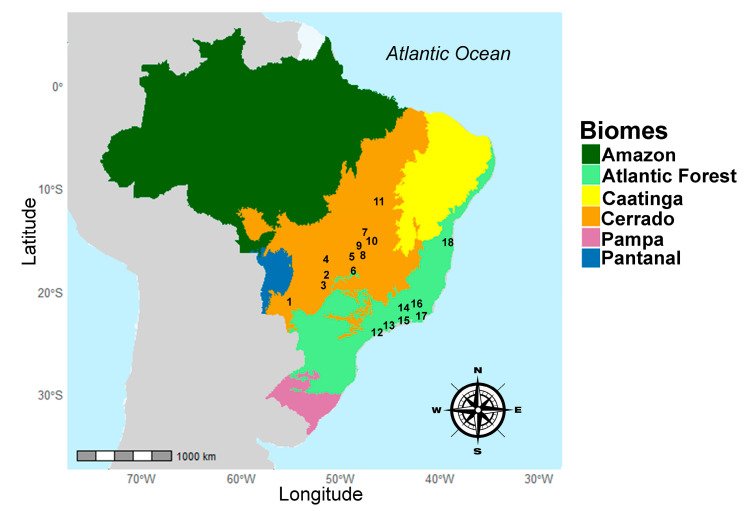
Geographical distribution of the 45 analyzed specimens of *O. catherinae*. Collection localities span eight Brazilian states. Cerrado biome: Distrito Federal: Brasília (9); Goiás: Anápolis (5), Aporé (3), Caiapônia (4), Caldas Novas (6), Luziânia (8), Niquelândia (7), Planaltina (10), Serranópolis (2); Mato Grosso do Sul: Dois Irmãos do Buriti (1); Tocantins: Dianópolis (11). Atlantic Forest biome: Bahia: Ilhéus (18); Minas Gerais: Pirapetinga (16); São Paulo: Ubatuba (12); Rio de Janeiro: Cachoeiras de Macacu (15), Casimiro de Abreu (17), Guapimirim (13), Sumidouro (14).

Our sampling encompassed 18 localities, distributed across two biomes (Figure 1), The first is the Atlantic Forest biome, including the states of Bahia (Ilhéus), Rio de Janeiro (Cachoeiras de Macacu, Casimiro de Abreu, Guapimirim, Sumidouro), and São Paulo (Ubatuba), presenting habitats ranging from coastal lowland forests to montane formations. The second is the Cerrado biome, including the state of Tocantins (Dianópolis), Goiás (Anápolis, Aporé, Caiapônia, Caldas Novas, Luziânia, Planaltina, Niquelândia, Serranópolis), Distrito Federal (Brasília), Minas Gerais (Pirapetinga), and Mato Grosso do Sul (Dois Irmãos do Buriti), covering vegetation types from open grasslands to forested savannas.

### 2.2. Age Classification and Morphometric Measurements

**Figure 2:**
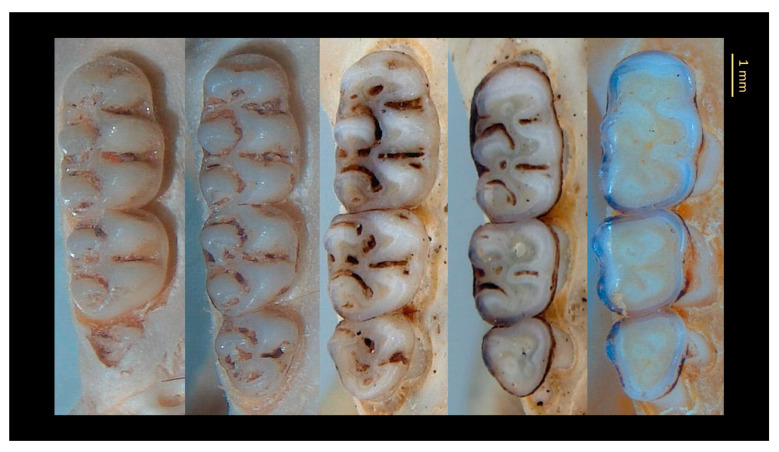
Molar dentition in *O. catherinae* showing stages of development and wear, from Classes 1 to 5, following [21] (from left to right, vouchers LBCE16562, CRB4023, CRB4025, MN33620, MN74360) considered in the age classification.

Specimens were classified into five age categories based on eruption stage and wear of the third molar (M3), following [21] (Figure 2, Table 1); only specimens from Classes 2 to 5 were included to avoid ontogenetic bias in morphometric analyses, excluding juveniles (Class 1).

**Figure 3:**
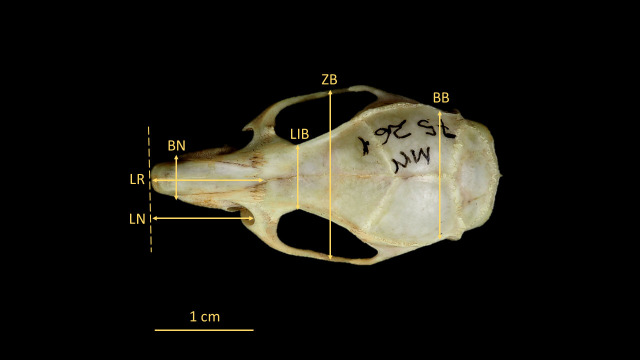
Skull of *O. catherinae* (MN75261) in dorsal view, following [21,22,23], showing cranial measurements: LN = length of nasals; LR = length of rostrum; BN = breadth of nasals; LIB = least interorbital breadth; ZB = zygomatic breadth; and BB = breadth of braincase.

**Figure 4:**
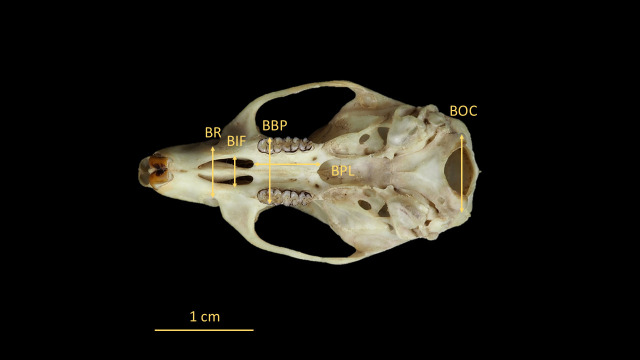
Skull of *O. catherinae* (MN75261) in ventral view, following [21,22,23], showing cranial measurements: BR = breadth of rostrum; BIF = breadth of incisive foramina; BBP = breadth across bony palate; BPL = bony palate length; and BOC = breadth of occipital condyles.

**Figure 5:**
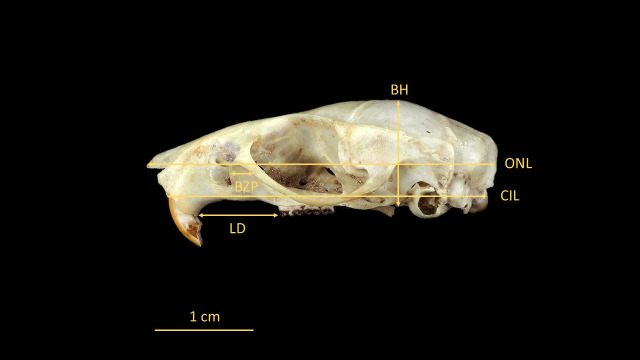
Skull of *O. catherinae* (MN75261) in lateral view, following [21,22,23], showing cranial measurements: LD = length of diastema; BZP = breadth of zygomatic plate; BH = braincase height; ONL = occipitonasal length; and CIL = condylo-incisive length.

**Figure 6:**
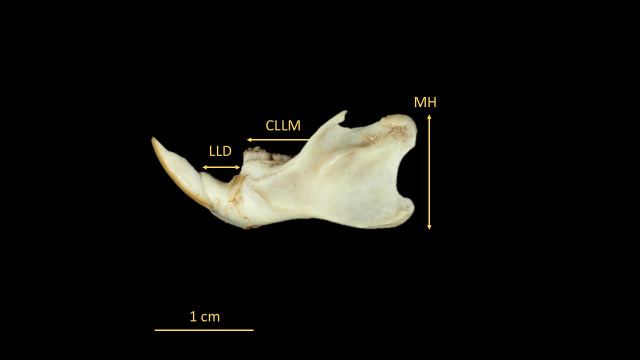
Mandible of *O. catherinae* (MN75261) in lateral view, following [21,22,23], showing measurements: LLD = lower length of diastema; CLLM = coronal length of lower molars; and MH = mandibular height.

**Figure 7:**
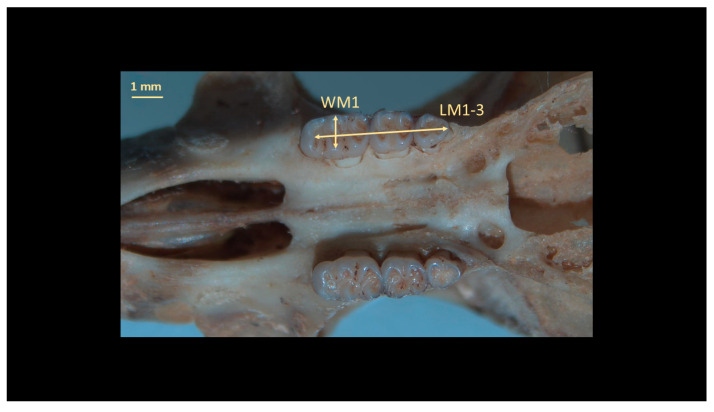
Upper dentition of *O. catherinae* (MN44805), following [21,22,23], showing measurements: WM1 = width of first upper molar (M1) and LM1—3 = coronal length of maxillary toothrow.

For the identification of *O. catherinae*, the key anatomical structures were measured following protocols derived from previous rodent studies [21,22,23] with methodological adaptations specific to *Oecomys* morphology [2]. Measurements were taken in dorsal, ventral, and lateral views using a ZAAS digital caliper (150 mm), recording 21 craniodental parameters (Figure 3, Figure 4, Figure 5, Figure 6 and Figure 7, Table 2, Table S2: Mean values and standard deviation (SD) of morphometric variables measured from specimens of *O. catherinae* collected in different locations of the Brazilian Atlantic Forest and Cerrado). All measurements were taken three times by the same observer, and the mean value was used in analyses.

### 2.3. Data Analysis

Sexual dimorphism was analyzed to verify morphometric differences between male and female specimens. This was performed using PERMANOVA (Permutational Multivariate Analysis of Variance), based on a Bray–Curtis distance matrix [24], calculated from the measured morphometric variables. This determined whether females and males could be pooled for more robust sampling of phenotypic variation analyses.

Interbiome variation between Cerrado (N = 19) and Atlantic Forest (N = 26) populations was examined using discriminant analysis of principal components (DAPC) [25]. DAPC was selected for its ability to maximize between-group variation while minimizing within-group variation [25]. The optimal number of principal components was determined via cross-validation [26]. The statistical significance of the difference between biomes was also tested with PERMANOVA on the morphometric distance matrix. Furthermore, the effect of latitude on the morphometric variation in the species was investigated using distance-based redundancy analysis (db-RDA), with significance assessed by permutation testing [27]. This analysis aimed to investigate whether there is an influence of a given latitudinal gradient on the morphometry of *O. catherinae*.

In PERMANOVA and db-RDA, the F-statistic is the ratio of explained to residual mean squares. Its first degree of freedom represents the number of parameters tested, while the second corresponds to the residual degrees of freedom (calculated as the number of observations minus the number of estimated parameters). R^2^ is the proportion of variance explained by the model, and the *p*-value is the probability of obtaining such an F under the null hypothesis [28].

Analyses were conducted in R v4.4.1 [29]. DAPC used the ‘adegenet’ package v2.1.10 [30]. PERMANOVA, distance matrices, permutation tests, and db-RDA employed the ‘vegan’ package v2.6-10 [31]. A 5% significance threshold was applied [32].

## 3. Results

### 3.1. Sexual Dimorphism

The PERMANOVA results revealed no statistically significant sexual dimorphism (R^2^ = 0.06; F_1,46_ = 2.73; *p* = 0.08), indicating that male and female *O. catherinae* do not exhibit significant differences in morphometric traits. Consequently, all specimens (males and females) were included in subsequent analyses.

### 3.2. Comparison Between Biome Populations

**Figure 8:**
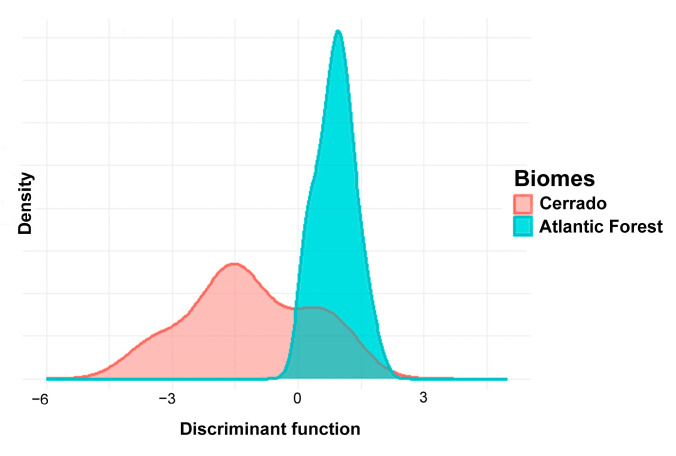
Graph of the discriminant analysis of principal components (DAPC) showing population groupings of *O. catherinae* specimens based on morphometric measurements across the Cerrado and Atlantic Forest biomes.

**Figure 9:**
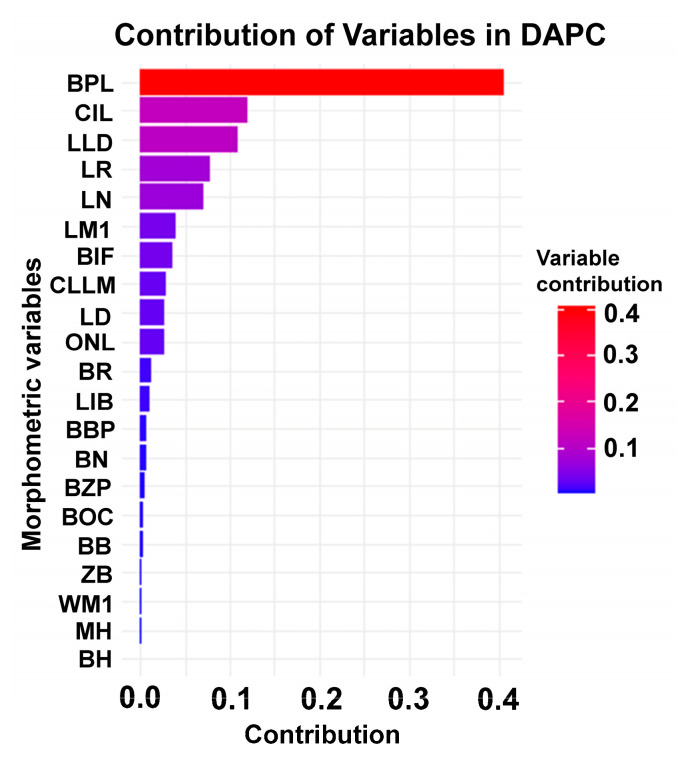
Contributions of morphometric variables to the separation of *O. catherinae* specimens between biomes (Cerrado and Atlantic Forest) in the discriminant analysis of principal components (DAPC).

DAPC comparing *O. catherinae* populations from the Cerrado and Atlantic Forest biomes retained six principal components, which explained approximately 96% of the total variation. The analysis correctly classified 72% of specimens into their respective biome groups. Significant morphometric differences were detected between the two biomes (R^2^ = 0.18; F_1,46_ = 10.36; *p* < 0.01; Figure 8). The analysis identified BPL as the most influential morphometric variable in distinguishing the groups (Figure 9).

### 3.3. Latitude Effect

**Figure 10:**
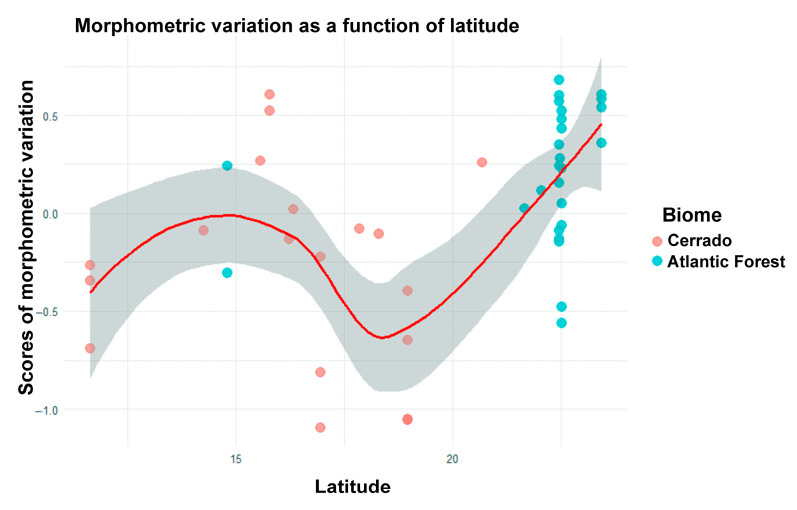
Relationship between the scores of the first canonical axis (CAP1) of the db-RDA analysis and the latitude of the specimen localities of *O. catherinae*. Each point represents an individual. The red line represents the trend curve adjusted by LOESS (Locally Estimated Scatterplot Smoothing), with a 95% confidence interval (shaded area). The increase in CAP1 scores with latitude suggests a morphological gradient in response to geographic variation.

A statistically significant, although weak, influence of latitude on the morphometric variation in *O. catherinae* was recorded (F_1,46_ = 3.63; adjusted R^2^ = 0.05; *p* = 0.03). This indicates that the morphometry of the specimens varies with latitude, with a general tendency to increase at higher latitudes (Figure 10). Specifically, specimens from southern localities (farther from the Equator) tended to exhibit larger morphometric measurements (e.g., cranial or dental dimensions), while those from northern localities (closer to the Equator) showed smaller values. However, this latitudinal pattern was non-linear, as evidenced by the scatterplot, indicating that the rate of morphometric change does not follow a consistent gradient across latitudes.

### 3.4. Oecomys catherinae Craniodental Morphometric Comparison Between Atlantic Forest and Cerrado Biomes

In general, skulls of *O. catherinae* from the Atlantic Forest tend to be wider and longer, while those from the Cerrado show a somewhat more reduced and variable morphology. In this regard, Atlantic Forest specimens presented slightly larger skulls, with higher mean values for characteristics such as bony palate length (BPL: 6.89 ± 0.46 mm vs. 6.03 ± 0.92 mm), condylo-incisive length (CIL: 28.83 ± 1.41 mm vs. 28.06 ± 2.34 mm), and length of nasals (LN: 11.54 ± 0.91 mm vs. 9.64 ± 2.16 mm). Additionally, the lower length of diastema (LLD) was also greater in the Atlantic Forest (3.03 ± 0.31 mm) compared to in the Cerrado (2.71 ± 0.53 mm). On the other hand, length of rostrum (LR) showed slightly higher mean values in Cerrado specimens (9.28 ± 1.60 mm) than in Atlantic Forest specimens (9.16 ± 0.74 mm) (Table S2: Mean values and standard deviation (SD) of morphometric variables measured in *O. catherinae* specimens from Atlantic Forest and Cerrado locations).

## 4. Discussion

The absence of significant sexual dimorphism in *O. catherinae* corroborates previously documented patterns for the genus [33,34] and may reflect selective pressures for locomotor efficiency in arboreal habitats, which stabilize traits associated with this mode of movement [35]. This pattern contrasts with terrestrial rodents, where intense male competition for access to females leads to pronounced sexual dimorphism [36]. Despite this intersexual conservatism, *O. catherinae* exhibits significant morphological variation among populations from different biomes. Our DAPC analysis revealed morphometric divergence between Atlantic Forest and Cerrado individuals, with detectable latitude effects suggesting phenotypic plasticity along ecological gradients. This pattern supports our hypothesis and mirrors findings in other neotropical rodents: *Holochilus brasiliensis* showed biome-associated morphometric differences linked to vegetation structure [37], while four species (*Akodon cursor*, *Cerradomys subflavus*, *Oligoryzomys nigripes*, and *Oxymycterus dasythrichus*) demonstrated cranial morphology shifts along temperature and humidity gradients [38]. These cases reinforce the fundamental role of environmental factors in shaping morphological changes in small mammals.

Although latitude showed only modest effects on morphometric variation, this pattern aligns with both Bergmann’s rule (which predicts larger body size at higher latitudes) and latitudinal diversity patterns [39]. These trends may reflect adaptations to climatic pressures or resource availability, with environmental humidity—through its effect on heat exchange—representing one potentially significant factor influencing mammalian body size variation [40]. The macroecological trends identified here, while consistent with theoretical predictions [41,42], require future studies combining morphological time series and in situ ecological data. Such a multidimensional approach would prove particularly valuable for assessing latitudinal variation, where morphological patterns may emerge from contemporary ecological pressures, evolutionary legacies, or their interactions—not only in *O. catherinae*, but as a model for neotropical small mammal diversification.

Smaller skulls in the Cerrado compared to in the Atlantic Forest may reflect thermal adaptation, aligning with the trend of size reduction in warmer climates [43]. The longer snout in Cerrado individuals could be an adaptation to drier climates, facilitating thermoregulation and reducing water loss, similarly to patterns observed in other rodents where humidity and temperature influence rostral development [44]. The greater morphological variability in the Cerrado may reflect an adaptive response to environmental heterogeneity and seasonal resource fluctuations, while the uniformity in the Atlantic Forest may be associated with more stable ecological conditions, since body size is shaped by multiple ecological pressures (e.g., resource availability vs. competition) that act in concert—studying them in isolation may lead to misinterpretations [45].

Skull shape differences may also relate to diet: in the Cerrado, a longer rostrum may improve foraging efficiency in an environment with more dispersed resources and harder seeds, while in the Atlantic Forest, a more predictable diet may favor more uniform skulls [46,47]. These morphological differences in *O. catherinae* between the Atlantic Forest and Cerrado follow patterns observed in other rodents, where climate, resource availability, and competition shape cranial morphology [38]. The smaller size and greater variability in the Cerrado may represent adaptive responses to a hotter, drier, and more heterogeneous environment, while the Atlantic Forest’s uniformity reflects more stable ecological conditions.

Our spatially representative sampling across Atlantic Forest and Cerrado biomes, combined with detailed morphological analyses, provides a robust foundation for macroevolutionary inferences (i.e., large-scale evolutionary patterns across time/space) [48,49]. However, as this study focused solely on cranial variation, locomotor effects remain speculative. Larger samples would enhance resolution for detecting subtle interpopulation variation, while integrating ecological data (e.g., microclimate measurements) with molecular data (e.g., selection signatures in candidate genes) could clarify whether observed variations reflect phenotypic plasticity, local adaptation, gene–environment interactions, or cryptic speciation.

To advance this research, three complementary approaches appear particularly promising: (1) application of biomechanical models [50] to test form–function relationships across different contexts; (2) genomic integration [51] to discriminate plastic vs. genetic components of variation; and (3) temporal analyses of museum specimens [52] to assess historical responses to environmental change. Additionally, future studies could investigate postcranial skeletal variation (e.g., limb proportions, pelvic morphology) to test hypotheses about locomotor efficiency and its role in biome-specific adaptations.

## 5. Conclusions

Our study reveals that *O. catherinae* exhibits no significant sexual dimorphism, consistent with stabilizing selection for arboreal locomotion, while significant cranial divergence exists between Atlantic Forest and Cerrado populations. The biome-specific morphological differences likely reflect adaptations to warmer, drier climates and a more heterogeneous resource distribution in the Cerrado compared to in the stable Atlantic Forest environment. The influence of latitude on cranial morphometry was modest but aligned with ecogeographic trends, such as Bergmann’s rule.

To fully disentangle the roles of phenotypic plasticity and genetic adaptation, future studies should integrate genomic analyses with biomechanical modeling. Investigating postcranial morphology could further clarify how locomotor specialization interacts with environmental pressures. These findings underscore the importance of biome-driven selection in shaping morphological diversity in neotropical rodents, offering a framework for understanding adaptive responses to habitat variation.

## Figures and Tables

**Table 1:** Age classification of *O. catherinae* specimens based on third molar (M3) eruption stage and occlusal wear patterns, following [21].

Age Classification	Description
Class 1 (juveniles)	M3 incompletely erupted or unworn.
Class 2 (adults)	M3 fully erupted exhibiting slight-to-moderate wear (some dentine exposed), but with the occlusal surface still tubercular (the paracone raised and prominent) not flat.
Class 3 (intermediate-stage adults)	M3 well worn and the occlusal surface flat or concave; M1-2 tubercular (the major cusps all separate and prominent); anteroloph of M2 distinct, not fused with paracone.
Class 4 (advanced-stage adults)	M3 flat or concave; M1-2 with cusps almost worn or quite flat but not below widest part of crown; anteroloph of M2 obliterated, fused with paracone.
Class 5 (senile adults)	MI-3 all worn flat or concave, below widest part of crowns; most details of occlusal topography obliterated.

**Table 2:** Craniometric measurements of *O. catherinae*, including abbreviations (Abb), descriptions, and anatomical definitions of cranial and mandibular measurements [21,22,23]. All measurements are given in millimeters (mm).

Abb	Measurement	Description
BH	Braincase Height	Braincase height maximum vertical extent of the internal cranial region, from the base to the highest point of the skull.
BB	Breadth of Braincase	Measured at the plane dorsal to the squamosal roots of the zygomatic arches and ventral to the temporal ridges.
LIB	Least Interorbital Breadth	The least distance across the frontal bones between the orbital fossae.
BBP	Breadth across Bony Palate	The greatest breadth across the basal part of the palate.
BIF	Breadth of the Incisive Foramina	The greatest inside breadth across both incisive foramina.
WM1	Width of the First Upper Molar (M1)	Coronal width of first upper molar.
BN	Breadth of Nasals	The greatest breadth across both nasal bones.
BR	Breadth of Rostrum	Measured just inside the anteroventral edge of the zygomatic plate.
LN	Length of Nasals	The greatest length of either nasal bone.
LR	Length of Rostrum	Longitudinal extent of the anterior or frontal part of the skull, including the facial bone region.
BZP	Breadth of the Zygomatic Plate	The least distance between anterior and posterior edges of the zy- gomatic plate.
BOC	Breadth of the Occipital Condyles	The greatest breadth across the dorsal lobes of both occipital condyles.
LM1-3	Coronal Length of Maxillary Toothrow	Measured along the alveolar margin from the anterior edge of M1 to the posterior edge of M3.
LD	Length of Diastema	Measured from the crown of the first maxillary molar to the exposed lesser curvature of the upper incisor on the same side.
BPL	Bony Palate Length	The linear distance from the posterior margin of the incisive foramen to the anteriormost point of the posterior palatal border.
CLLM	Coronal Length of Lower Molars	Linear length of the crown of the lower molars (M1–M3).
LLD	Lower Length of Diastema	Linear distance of the toothless gap between the incisors and molars in the lower dentition.
MH	Mandibular Height	Maximum vertical extent of the mandible.
CIL	Condylo-Incisive Length	Measured from the exposed greater curvature of an upper incisor to the articular surface of the occipital condyle on the same side.
ONL	Occipitonasal Length	Greatest length of skull, from the anteriormost projection of the nasal bones to the posteriormost projection of the occipital bone.
ZB	Zygomatic Breadth	The greatest breadth across the zygomatic processes of the squamosal bones.

## Data Availability

The data supporting the findings of this study are included within the article (Supplementary Files). No new external data were generated.

## Supplementary Materials

The following supporting information can be downloaded at https://www.mdpi.com/article/10.3390/ani15152200/s1, Table S1: Geographic location, sex, and age classification of *O. catherinae* specimens; Table S2: Mean values and standard deviation (SD) of morphometric variables measured from specimens of *O. catherinae* collected in different locations of the Brazilian Atlantic Forest and Cerrado.

## Abbreviations

The following abbreviations are used in this manuscript:

MN	Museu Nacional
UFRJ	Universidade Federal do Rio de Janeiro
COLMASTO	Coleção Integrada de Mamíferos Reservatórios Silvestres
IOC	Instituto Oswaldo Cruz
FIOCRUZ	Fundação Oswaldo Cruz
LBCE	Laboratório de Biologia e Controle da Esquistossomose
CRB	Cibele Rodrigues Bonvicino
BA	Bahia
GO	Goiás
MG	Minas Gerais
MS	Mato Grosso do Sul
RJ	Rio de Janeiro
SP	São Paulo
TO	Tocantins
DF	Distrito Federal
M3	Third Molar
M1	First Molar
LN	Length of Nasals
LR	Length of Rostrum
BN	Breadth of Nasals
BR	Breadth of Rostrum
LIB	Least Interorbital Breadth
ZB	Zygomatic Breadth
BB	Breadth of Braincase
BIF	Breadth of the Incisive Foramina
BPL	Bony Palate Length
BBP	Breadth across Bony Palate
BOC	Breadth of the Occipital Condyles
LD	Length of Diastema
BZP	Breadth of the Zygomatic Plate
BH	Braincase Height
ONL	Occipitonasal Length
CIL	Condylo-Incisive Length
LLD	Lower Length of Diastema
CLLM	Coronal Length of Lower Molars
MH	Mandibular Height
LM1-3	Coronal Length of Maxillary Toothrow
WM1	Width of the First Upper Molar (M1)
SD	Standard Deviation
PERMANOVA	Permutational Multivariate Analysis of Variance
DAPC	Discriminant Analysis of Principal Components
db-RDA	Distance-Based Redundancy Analysis
MANOVA	Multivariate Analysis of Variance
CAP1	First Canonical Axis
