# Supplementary material for: Phenotypic Variation Patterns in Oecomys catherinae (Rodentia: Sigmodontinae): Craniodental Morphometric Analysis and Its Relationship with Latitudinal Variation in the Atlantic Forest and Cerrado Biomes
Source: Animals (Basel). 2025 Jul 26;15(15):2200. doi: 10.3390/ani15152200 (PMC12345570; doi:10.3390/ani15152200)
Supplement: Supplementary file 1 [file animals-15-02200-s001.zip › animals-3722931_TableS2.pdf]

Table S2. Mean values and standard deviation (SD) of morphometric variables measured from specimens of *O. catherinae* collected in different locations of the Brazilian Atlantic Forest and Cerrado.

Morphometric variable	Description	Biome			
		Atlantic Forest		Cerrado	
		Mean	SD	Mean	SD
BB	Breadth of Braincase	12.791	0.398	12.237	0.516
BBP	Breadth across Bony Palate	6.107	0.257	5.810	0.499
BH	Braincase Height	9.408	0.398	9.173	0.436
BIF	Breadth of the Incisive Foramina	2.837	0.208	2.996	0.481
WM1	Width of the first upper Molar (M1)	1.548	0.090	1.390	0.195
BN	Breadth of Nasals	3.863	0.365	3.812	1.253
BOC	Breadth of the Occipital Condyles	10.918	0.640	9.722	1.677
BR	Rostrum width	6.336	0.466	5.791	1.316
BZP	Breadth of the Zygomatic Plate	3.714	0.304	3.409	0.513
CIL	Condylo-Incisive Length	28.874	1.395	27.885	2.304
CLLM	Coronal Length of Lower Molars	5.200	0.146	4.922	0.468
LM1-3	Coronal Length of Maxillary toothrow	5.118	0.174	4.774	0.507
LD	Length of Diastema	8.413	0.688	7.860	0.660
LIB	Least Interorbital Breadth	5.999	0.413	5.560	0.437
LLD	Lower Length of Diastema	3.017	0.312	2.709	0.503
LN	Length of Nasals	11.561	0.904	9.427	2.169
BPL	Bony Palate Length	6.897	0.449	5.920	0.956
LR	Length of Rostrum	9.166	0.727	9.320	1.530
MH	Mandibular Height	8.511	0.615	7.813	1.049
ONL	Occipitonasal Length	32.970	1.650	30.871	2.673
ZB	Zygomatic Breadth	17.205	1.076	15.893	1.309
